# FLEX-SFL: A Flexible and Efficient Split Federated Learning Framework for Edge Heterogeneity

**DOI:** 10.3390/s25206355

**Published:** 2025-10-14

**Authors:** Hao Yu, Jing Fan, Hua Dong, Yadong Jin, Enkang Xi, Yihang Sun

**Affiliations:** 1 School of Electrical and Information Technology, Yunnan Minzu University, Kunming 650504, China; haoyu531@yeah.net (H.Y.); dh1643694780@163.com (H.D.); jinyadong131468@163.com (Y.J.); cek15216379868@163.com (E.X.); sunyihang0311@163.com (Y.S.); 2 Yunnan Key Laboratory of Unmanned Autonomous System, Yunnan Minzu University, Kunming 650504, China; 3 University Key Laboratory of Information and Communication on Security Backup and Recovery in Yunnan Minzu University, Kunming 650504, China

**Keywords:** federated learning (FL), split learning (SL), edge heterogeneity, client selection, asynchronous aggregation

## Abstract

**Highlights:**

**What are the main findings?**

- The FLEX-SFL framework introduces dynamic, device-aware adaptive model segmentation, entropy-driven client selection, and hierarchical local asynchronous aggregation mechanisms, improving training efficiency and scalability in edge heterogeneous environments.
- Extensive experiments demonstrate that FLEX-SFL outperforms state-of-the-art federated and split federated learning methods in terms of accuracy, convergence speed, and resource efficiency across multiple datasets.

**What are the implications of the main findings?**

- FLEX-SFL provides a practical solution to the challenges posed by system and statistical heterogeneity in federated learning, making it suitable for large-scale edge deployments in real-world intelligent systems.
- The proposed mechanisms can be extended to enhance the scalability and adaptability of other federated learning frameworks, potentially improving edge computing applications in fields like IoT and healthcare.

**Abstract:**

The deployment of Federated Learning (FL) in edge environments is often impeded by system heterogeneity, non-independent and identically distributed (non-IID) data, and constrained communication resources, which collectively hinder training efficiency and scalability. To address these challenges, this paper presents FLEX-SFL, a flexible and efficient split federated learning framework that jointly optimizes model partitioning, client selection, and communication scheduling. FLEX-SFL incorporates three coordinated mechanisms: a device-aware adaptive segmentation strategy that dynamically adjusts model partition points based on client computational capacity to mitigate straggler effects; an entropy-driven client selection algorithm that promotes data representativeness by leveraging label distribution entropy; and a hierarchical local asynchronous aggregation scheme that enables asynchronous intra-cluster and inter-cluster model updates to improve training throughput and reduce communication latency. We theoretically establish the convergence properties of FLEX-SFL under convex settings and analyze the influence of local update frequency and client participation on convergence bounds. Extensive experiments on benchmark datasets including FMNIST, CIFAR-10, and CIFAR-100 demonstrate that FLEX-SFL consistently outperforms state-of-the-art FL and split FL baselines in terms of model accuracy, convergence speed, and resource efficiency, particularly under high degrees of statistical and system heterogeneity. These results validate the effectiveness and practicality of FLEX-SFL for real-world edge intelligent systems.

## 1. Introduction

The rapid proliferation of intelligent edge terminals, along with the increasing demand for data privacy and real-time decision-making, has led to the rise of collaborative distributed learning as a key enabler for the evolution of edge intelligence in sensor networks and IoT systems [1]. Federated Learning (FL), a prominent privacy-preserving collaborative learning paradigm, facilitates the joint training of a global model across distributed devices without requiring the transfer of raw data, thus enhancing privacy protection. However, FL faces two major challenges in practical deployment, particularly in sensor networks. The first, is system heterogeneity, where edge devices exhibit significant variations in terms of computational power, storage capacity, and network bandwidth, leading to inefficiencies in training and model aggregation. The second is statistical heterogeneity, where the data collected by devices is often non-independent and identically distributed (non-IID), causing slow convergence and unstable performance, which are critical issues in sensor-driven environments where data diversity is high [2]. These challenges significantly hinder the scalability and effectiveness of FL in large-scale edge networks, such as those commonly found in sensor and IoT systems.

To address these issues, Split Learning (SL) has been introduced, which partitions deep learning models between clients and servers, helping to reduce the computational burden on resource-constrained edge devices. While SL improves the participation of low-performance devices, its reliance on serial communication and lack of parallel optimization limits its ability to support large-scale, multi-terminal collaboration. As a result, Split Federated Learning (SFL), which combines the strengths of both FL and SL, has emerged as a promising solution. SFL aims to optimize model partitioning and federated aggregation while ensuring data privacy across diverse devices [3]. Despite its potential, existing SFL approaches are hindered by limitations such as static model partitioning, inadequate client selection strategies, and heavy synchronization dependencies in the training process, which compromise their adaptability and efficiency in highly heterogeneous edge environments, like those seen in sensor networks [4].

To address the above issues, this paper proposes a flexible and efficient federated split learning framework for edge heterogeneous environments, named FLEX-SFL (Flexible and Efficient Split Federated Learning), tailored for heterogeneous edge environments. The framework integrates three complementary mechanisms focusing on adaptive model partitioning, representative client participation, and efficient hierarchical aggregation:

(1) Device-aware adaptive model segmentation (DAS): This mechanism dynamically determines the optimal model split point for each client based on its computing and communication capabilities, mitigating the “straggler” effect and improving resource utilization;

(2) Entropy-driven client selection strategy (EDCS): This mechanism measures data representativeness via label-distribution entropy and applies a lightweight heuristic selection algorithm to enhance data diversity and global generalization;

(3) Hierarchical local asynchronous aggregation mechanism (HiLo-Agg): This mechanism employs a two-level aggregation structure that decouples local client updates from global synchronization, effectively reducing communication delay and accelerating convergence.

The main contributions of this paper are as follows:

- A unified flexible Split Federated Learning framework (FLEX-SFL) is proposed to jointly address system heterogeneity, statistical heterogeneity, and communication bottlenecks in edge environments;
- An adaptive segmentation mechanism (DAS) is designed to personalize model partitioning according to each client’s resource profile, enhancing scalability and training efficiency;
- An entropy-driven client selection strategy (EDCS) is introduced to achieve better balance between representativeness and efficiency under non-IID data;
- A hierarchical local asynchronous aggregation mechanism (HiLo-Agg) is developed to enable asynchronous multi-level aggregation, alleviating synchronization delays while preserving global model consistency;
- Comprehensive experiments on multiple non-IID benchmarks (FMNIST, CIFAR-10, CIFAR-100) verify that FLEX-SFL achieves superior accuracy, faster convergence, and lower communication cost compared with state-of-the-art SFL methods.

FLEX-SFL provides a practical and generalizable framework for building adaptive and communication-efficient edge intelligence systems, with promising potential for real-world IoT deployments.

## 2. Related Work

This section reviews the main research paradigms and representative works in the field of federated intelligence around the core technical directions of this paper, with a focus on FL, SL, and their fusion SFL, to lay the foundation for the proposed FLEX-SFL framework.

### 2.1. Federated Learning

**Figure 1:**
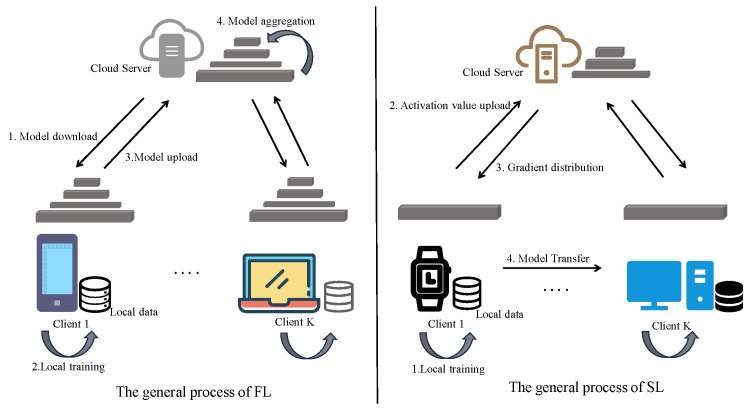
The general processes of federated learning and split learning.

FL is a typical distributed collaborative learning framework. Its core principle is that clients train submodels on local data without centralizing data, and only upload model gradients or parameters to the server for aggregation, as shown on the left side of Figure 1 for its general process. This approach effectively alleviates data silos and privacy leakage issues, and is widely applied in scenarios such as healthcare [5], finance [6], and smart terminals [7]. Its standard optimization objective can be formalized as

(1) $$\underset{W}{\min}\sum_{k=1}^{K}p_{k}F_{k}(w)$$

where W is the global model parameter, $F_{k}(w)$ represents the loss function of the k-th client, K is the total number of clients, and $p_{k}$ is the data proportion of client k.

Despite FL’s strong privacy protection and system scalability, it faces two key challenges in practical deployment:

- System heterogeneity: Significant variations in computing power, energy consumption, storage, and communication quality among clients often cause low-performance devices to slow down the overall training process, leading to the “straggler effect.”
- Statistical heterogeneity: Data distributions across clients generally exhibit non-independent and identically distributed (non-IID) characteristics, making local models prone to bias and affecting the convergence stability and generalization ability of the global model.

To address these challenges, academia has proposed a series of optimization methods. For statistical heterogeneity, Li et al. [8] proposed FedProx, which introduces a regularization term into the local optimization objective to limit model drift; Mora and Zhou et al. [9,10] mitigated non-IID effects by constructing a proxy dataset on the server side for post-training recalibration of local models. For system heterogeneity, FedBuff and AFL adopted asynchronous update mechanisms to improve training parallelism [11,12]; Maciel et al. [13] pre-screened participable devices based on client status (e.g., battery level and connection quality); Zhu and Zhao et al. [14,15] minimized waiting time and energy consumption through bandwidth scheduling strategies.

Although the above methods have achieved optimization effects in specific dimensions, most focus on a single heterogeneous factor and lack a unified mechanism for collaborative optimization of system resources, data distribution, and communication efficiency, making them difficult to adapt to the high dynamics and heterogeneity of real-world edge scenarios.

### 2.2. Split Learning

Furthermore, with the rapid proliferation of smart terminals (e.g., smartwatches, wristbands, wearable devices) and the substantial increase in task complexity and model parameter scale, edge devices face ever-growing computational and communication burdens when participating in deep learning model training [16]. For instance, ResNet18, ResNet50, and ResNet101 contain approximately 11.2 million, 25.6 million, and 44.5 million parameters, respectively, rendering full-model training unfeasible for resource-constrained devices [17]. He et al. demonstrated that FL exhibits superior communication efficiency when the number of clients is small or the model size is limited; however, in scenarios with a large client base or high model complexity, FL’s communication overhead escalates rapidly, leading to a marked decrease in efficiency.

To alleviate the participation barriers for lightweight devices, Gupta et al. [18] proposed the SL paradigm, whose general process is illustrated on the right side of Figure 1. The core principle involves partitioning deep models into client-side and server-side segments: clients are responsible for performing forward propagation and uploading activation values, while the server manages backward propagation and parameter updates. This mechanism significantly reduces terminal computational and storage pressures through structural computation offloading, making it suitable for edge devices deploying deep models.

However, SL inherently relies on a serial communication architecture, necessitating sequential interaction between multiple clients and the server. This lack of parallelism in the training process renders it inadequate for supporting large-scale multi-client concurrent scenarios. Additionally, due to its fundamental nature as a single-device or non-federated architecture, SL demonstrates limited capability in handling multi-source non-IID data distributions and lacks cross-client modeling capabilities. Consequently, while SL excels in adapting to lightweight devices, its generalization ability, communication efficiency, and collaborative training capacity remain insufficient to meet the practical demands of complex edge systems [19].

### 2.3. Split Federated Learning

To simultaneously leverage the collaborative training capabilities of federated learning and the resource decoupling features of split learning, Split Federated Learning (SFL) has emerged as a critical development direction in distributed intelligent modeling [20]. In this paradigm, the model is partitioned into client-side submodels and server-side submodels, allowing multiple clients to participate in training in parallel while the server employs federated aggregation strategies to integrate uploaded information from all parties. This approach ensures data privacy while achieving friendly adaptation to resource-constrained devices. SFL reduces terminal load through structural partitioning and enhances model generalization performance via aggregation mechanisms, demonstrating significant engineering deployment potential.

**Figure 2:**
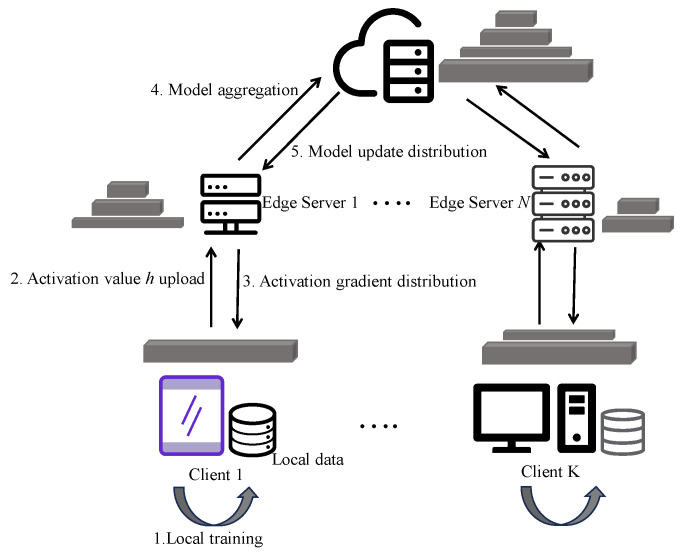
The general process of split federated learning.

Figure 2 illustrates the basic training workflow of SFL. Taking a typical deep neural network as an example, the model is divided into a client-side submodel $w_{c}$ and a server-side submodel $w_{s}$. The complete training process consists of four steps: (1) the client receives local input samples x and computes intermediate activation values $h=f_{c}(x;w_{c})$; (2) h is transmitted to the server, which performs $\hat{y}=f_{s}(h;w_{s})$ and calculates the loss L; (3) the server performs backpropagation based on the loss to update $w_{s}$ and sends the gradient ∂h back to the client; (4) the client uses this gradient to continue backpropagation and update $w_{c}$.

To enhance SFL’s adaptability and communication efficiency, numerous studies have expanded and optimized its training mechanisms. The SplitFed series, as a representative work, first proposed the “dual-side aggregation” strategy (SFL-V1), which synchronously aggregates client-side and server-side models. However, this method relies heavily on full-synchronous updates across all devices, leading to high communication latency. The subsequent SFL-V2 introduced an asynchronous mechanism, significantly improving training stability and delay tolerance [21].

Building on these foundations, researchers have explored optimizations in model structure design and aggregation mechanisms. SplitMix proposed a tunable partitioning mechanism that allows clients to select model splitting points according to their resource conditions, enhancing flexibility but lacking adaptive coordination across clients [22]. Cluster-HSFL introduced a cluster-based hierarchical training strategy, effectively alleviating the communication load of central servers, yet the static clustering configuration limited responsiveness to dynamic device variations [23]. FedLite employs Product Quantization (PQ) to compress activations and a gradient correction module to reduce quantization errors, thereby improving communication efficiency; however, model accuracy degraded under highly non-IID data distributions [24]. HSFL utilizes a Multi-Armed Bandit (MAB-BC-BN2) strategy that dynamically selected clients based on channel quality and local update magnitude, which improved resource utilization but introduced non-negligible control overhead [25]. CHEESE incorporates a helper–client mechanism that divides models into smaller subsegments for low-capability devices and adopts a ring topology for asynchronous collaboration, improving inclusiveness but occasionally leading to unbalanced convergence across clients. FedCST combines pruning and clustering strategies to mitigate training fluctuations caused by unstable client participation; however, aggressive pruning can reduce the model’s representation capacity [26].

While the above methods have achieved certain improvements in model flexibility and communication efficiency, existing SFL solutions still face three prominent issues in large-scale edge heterogeneous scenarios:

- Model partitioning remains predominantly static, lacking dynamic adaptive mechanisms based on device capabilities, which hinders personalized training efficiency;
- Most client selection strategies are random or round-robin, failing to effectively measure data representativeness and restricting global model training;
- Synchronous communication structures limit training concurrency, prone to blocking in weak-connection or high-latency devices, and reducing system throughput.

Although some studies have attempted to alleviate these issues through asynchronous communication, personalized modeling, distillation, and structural alignment, a universally applicable optimization framework with clear architecture, coordinated mechanisms, and the simultaneous addressing of system and statistical heterogeneities remains lacking.

## 3. System Framework and Design Scheme

**Figure 3:**
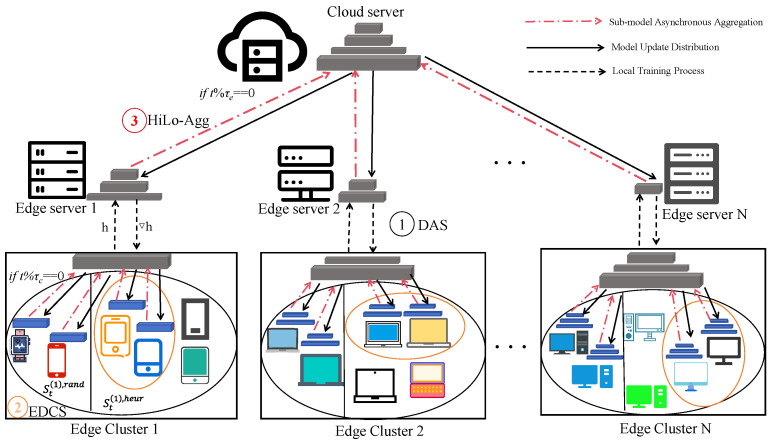
Schematic diagram of the FLEX-SFL framework.

To address the triple challenges of system heterogeneity, statistical heterogeneity, and communication overhead in edge intelligent systems, this paper proposes FLEX-SFL (Flexible and Efficient Split Federated Learning), a flexible and efficient optimization framework for split federated learning. Centering on three key issues—model partitioning, client selection, and model aggregation—the framework includes three core mechanisms: device-aware adaptive segmentation (DAS), entropy-driven client selection (EDCS), and hierarchical local asynchronous aggregation (HiLo-Agg). These mechanisms collaborate to achieve system resource adaptation, data representativeness enhancement, and communication efficiency improvement. The overall framework is illustrated in Figure 3.

In FLEX-SFL, the DAS module dynamically determines model partition points for each client based on its computing power status and aggregates devices with consistent partitioning structures into the same edge cluster, ensuring structural uniformity for personalized modeling and intra-cluster aggregation. The EDCS mechanism employs a “two-stage selection” strategy: first, random sampling to ensure device diversity, followed by heuristic screening based on label entropy to enhance the representativeness of selected clients’ data distributions. HiLo-Agg adopts a local–global dual-layer asynchronous aggregation architecture, enabling local asynchronous updates between clients and edge servers, and global aggregation between edge and central servers. This breaks through the synchronous communication bottleneck and enhances training throughput and system robustness. The main symbols used in this paper and their meanings are listed in Table 1.

### 3.1. Device-Aware Adaptive Segmentation

In practical deployments of FL, edge devices exhibit significant disparities in computing capabilities, where low-performance nodes often become the “performance bottleneck” of the system, forcing an extension of the training cycle. To mitigate this “straggler effect,” FLEX-SFL introduces DAS, a dynamic model partitioning strategy based on computing power and latency awareness. Its core objective is to adjust model partition positions on demand according to device capabilities, thereby achieving balanced allocation of training loads.

#### 3.1.1. Optimization Problem Modeling and Solution

Consider a system with K clients forming the set $C=\left\{C_{1},C_{2},\ldots ,C_{K}\right\}$, where the computing capability of the k-th client is denoted as $R_{k}$. In our implementation, the computing capability $R_{k}$ of each client is pre-determined based on its CPU floating-point performance, which remains fixed during training to reflect the static hardware heterogeneity across devices. The deep model to be trained consists of V layers, which is partitioned into a client-side submodel $w_{c}^{k}$ (comprising the first $p_{k}$ layers) and an edge server submodel $w_{e}^{k}$ (comprising layers $p_{k}+1$ to V).

The total delay of one training round primarily consists of three components: client-side forward propagation, activation value upload, and backpropagation, with edge server computation delay being negligible. Therefore, we model the training delay optimization problem as minimizing the maximum training time across the system:

(2) $$\min\;\max_{k\in C}\left(\frac{\delta_{k}\cdot C_{\mathrm{total}}}{R_{k}}\right)$$

where $\delta_{k}$ represents the proportion of training computation undertaken by client k, and $C_{\mathrm{total}}$ is the total computation of the full model. By introducing an auxiliary variable r, the above Min-Max problem can be transformed into the following linear programming form:

(3) minr

(3a) $$s.\;t.\;\frac{\delta_{k}C_{total}}{R_{k}}\leq r,\forall k$$

(3b) $$\sum_{k=1}^{K}\delta_{k}=1$$

Solving this programming problem yields the optimal computation proportion:

(4) $$\left\{\begin{array}{l} \delta_{k}^{*}=\frac{R_{k}}{\sum_{j=1}^{K}R_{j}}\\ s_{k}^{*}=\left\lfloor \frac{R_{k}}{\sum_{j=1}^{K}R_{j}}\cdot V\right\rfloor \end{array}\right.$$

where $s_{k}^{*}$ is the optimal model partition point for client $C_{k}$. This strategy achieves personalized allocation of training tasks based on device computing power, significantly enhancing the overall throughput of system training.

#### 3.1.2. Homogeneous Aggregation-Based Edge Cluster Partitioning Mechanism

Although DAS enables dynamic model partitioning according to device heterogeneity, personalized model structures introduce a critical issue: inconsistent submodel parameter dimensions across clients, which prevents direct model parameter aggregation and affects the consistency and convergence stability of the global model [27].

To address this heterogeneous aggregation barrier, FLEX-SFL designs an edge cluster partitioning mechanism. This mechanism aims to retain the flexible partitioning advantages of DAS while grouping clients with identical or similar partitioning structures into the same edge cluster. Edge servers within each cluster are responsible for corresponding submodel training and local aggregation, thereby achieving model structure consistency at the local level and ensuring aggregation stability.

Let the initial partition point set be $P=\left\{p_{1},p_{2},\ldots ,p_{K}\right\}$, where $p_{k}$ represents the original model partition point of client $C_{k}$. Denote the maximum and minimum values in this set as

(5) $$\left\{\begin{array}{l} p_{\max}=\max(P)\\ p_{\min}=\min(P) \end{array}\right.$$

Assume N edge servers are deployed in the system (i.e., supporting the creation of N edge clusters). The interval $\left[p_{\min},p_{\max}\right]$ is uniformly divided into N non-overlapping subintervals, with each interval width Δ defined as

(6) $$\Delta =\left\lceil \frac{p_{\max}-p_{\min}}{N}\right\rceil$$

The n-th subinterval is defined as

(7) $$I_{n}=\left[p_{\min}+(n-1)\Delta ,p_{\min}+n\Delta \right),\;n=1,2,\ldots ,N$$

For any client $C_{k}$, its partition point $p_{k}$ is assigned to subinterval $I_{n}$ and the distances to the upper and lower bounds of this interval are calculated as

(8) $$\left\{\begin{array}{l} d^{-}=|p_{k}-(p_{\min}+(n-1)\Delta )|\\ d^{+}=|p_{k}-(p_{\min}+n\Delta )|\; \end{array}\right.$$

According to the “minimum distance principle”, $p_{k}$ is discretized to the nearest boundary value to obtain the final partition point:

(9) $$p_{k}^{\mathrm{final}}=\arg\;\min\{d^{-},d^{+}\}$$

Based on this, the final partition point set is defined as $P^{final}=\{p_{1}^{\mathrm{final}},p_{2}^{\mathrm{final}},\ldots ,p_{K}^{\mathrm{final}}\}$. Clients with the same final partition point are assigned to the same edge cluster:

(10) $$E_{n}=\left\{C_{k}|p_{k}^{\mathrm{final}\;}=p_{\min}+(n-1)\Delta \;\mathrm{or}\;p_{\min}+n\Delta \right\}$$

This mechanism ensures that clients within each edge cluster have consistent submodel structures, facilitating local training and parameter aggregation within clusters. It also supports asynchronous parallel collaboration across clusters, effectively balancing model flexibility and aggregation feasibility. The device-aware adaptive segmentation (DAS) process is outlined in Algorithm 1.
**Algorithm 1.** DAS**Input:** Client partition point set P, number of edge server N
**Output:** Final discrete partition point set $P^{final}$ for each client, edge cluster partition result $\left\{E_{1},E_{2},\ldots ,E_{N}\right\}$
1: Calculate the maximum and minimum partition points $p_{\max}$ and $p_{\min}$
2: Compute the interval width Δ using Equation (6)3: Initialize $P^{final}=\emptyset$
4: for each client $C_{k}\in C$ do5:     Determine the subinterval $I_{n}$ containing the original partition point $p_{k}$
6:     Calculate $d^{-}$ and $d^{+}$ using Equation (8), discretize the partition point $p_{k}^{\mathrm{final}\;}$ using Equation (9)7:     Add $p_{k}^{\mathrm{final}\;}$ to $P^{\mathrm{final}}$
8: end for9: Construct the edge cluster $E_{n}$ set using Equation (10)10: return $P^{\mathrm{final}}$ and $\left\{E_{1},E_{2},\ldots ,E_{N}\right\}$


### 3.2. Entropy-Driven Client Selection for Data Heterogeneity

In FL, significant differences in data distributions among clients often lead to inconsistent update directions of local models, affecting the convergence and generalization ability of the global model. The “client drift” problem caused by this statistical heterogeneity is a critical bottleneck restricting system performance.

To address this issue, FLEX-SFL includes an entropy-driven client selection (EDCS) mechanism, which uses label information entropy as a metric for data diversity and combines a “random + heuristic” strategy to effectively improve the representativeness of client selection and enhance the stability and efficiency of model training.

#### 3.2.1. Label Entropy Modeling and Diversity Measurement

Consider an edge cluster $E_{n}$ containing a client set $C_{n}\subseteq C$. The label count vector of client k is

(11) $$n_{k}=[n_{k,1},n_{k,2},\ldots ,n_{k,Z}]\in {\mathbb{N}}^{Z}$$

where $n_{k,z}$ represents the number of samples in the z-th class, and Z is the total number of classes. The corresponding label distribution (probability vector) is

(12) $$\pi_{k}=\left[\frac{n_{k,1}}{N_{k}},\frac{n_{k,1}}{N_{k}},\ldots ,\frac{n_{k,Z}}{N_{k}}\right]$$

where $N_{k}=\overset{Z}{\underset{z=1}{\sum }}n_{k,z}$ is the total number of samples of client k. The information entropy (label diversity score) of client k is defined as

(13) $$H_{k}=-\sum_{z=1}^{Z}\pi_{k,z}\log(\pi_{k,z}+\varepsilon )$$

where ε is a constant to prevent logarithmic singularity, typically set to $10^{-8}$.

#### 3.2.2. Two-Stage Client Selection Process

To mitigate the bias effect caused by data imbalance, the optimization goal of this strategy is to select a subset from the candidate client set such that the label distribution of the subset is as close as possible to the global data distribution, i.e.,

(14) $$\underset{S_{t}^{\left(n\right)}\subseteq C_{n}}{\min}\mathrm{KL}\left(P_{t}^{\left(n\right)}∥P_{g}\right)$$

where $S_{t}^{\left(n\right)}$ denotes the selected client set, $P_{t}^{\left(n\right)}$ is the data distribution of the selected clients, $P_{g}$ is the global distribution, and KL(⋅∥⋅) represents the KL divergence.

In the t-th training round, the goal is to select a subset $S_{t}^{\left(n\right)}$ from $C_{n}$ to improve the label coverage of the selected set. The total selection size is controlled by the global participation rate ρ:

(15) $$K_{n}^{t}=\left\lceil \rho \cdot |C_{n}|\right\rceil$$

1. First Stage: Random Selection

Let the random sampling ratio be λ∈(0,1]. First, randomly select $K_{n}^{t,\mathrm{rand}}=\left\lceil \lambda \cdot K_{n}^{t}\right\rceil$ clients from $C_{n}$ to form an initial set: $S_{t}^{(n),\mathrm{rand}}\in C_{n}$, $|S_{t}^{(n),\mathrm{rand}}|=K_{n}^{t,\mathrm{rand}}$. The remaining candidate set is $S_{t}^{(n),\mathrm{rem}}=C_{n}\backslash S_{t}^{(n),\mathrm{rand}}$.

2. Second Stage: Greedy Entropy-Driven Selection

Select an additional $K_{n}^{t,\mathrm{rem}}=K_{n}^{t}-K_{n}^{t,\mathrm{rand}}$ clients from $S_{t}^{(n),\mathrm{rem}}$ using information entropy improvement as the heuristic criterion. Define the accumulated label vector of the selected clients as

(16) $$n_{\mathrm{sum}}=\sum_{k\in S_{t}^{(n),\mathrm{rand}}}n_{c}$$

For each candidate client $k^{\prime }\in S_{t}^{(n),\mathrm{rem}}$:

(17) $$n_{\mathrm{new}}=n_{\mathrm{sum}}+n_{k^{\prime }}$$

The proportion of the z-th class samples in the merged vector is

(18) $$\pi_{z}^{\mathrm{new}}=\frac{n_{z}^{\mathrm{new}}}{\sum_{j=1}^{Z}n_{j}^{\mathrm{new}}}$$

The merged label entropy is

(19) $$H^{\mathrm{new}}=-\sum_{z=1}^{Z}\pi_{z}^{\mathrm{new}}\log(\pi_{z}^{\mathrm{new}}+\varepsilon )$$

In each iteration, the client with the maximum $H^{\mathrm{new}}$ is selected to join the final set until $K_{n}^{t,\mathrm{rem}}$ clients are selected. The final selected set is

(20) $$S_{t}^{\left(n\right)}=S_{t}^{(n),\mathrm{rand}}\cup S_{t}^{(n),\mathrm{heur}}$$

The complexity of the random selection stage is $O(K_{n}^{t})$. For the greedy selection stage, since the entropy change of at most $\left|S_{t}^{(n),\mathrm{rem}}\right|$ clients (each with a z-dimensional vector entropy) is calculated in each iteration, and $K_{n}^{t,\mathrm{rem}}$ clients need to be selected, the total complexity is approximately

(21) $$O(\rho K(1-\lambda )\cdot K\cdot Z)\approx O(K^{2}Z)$$

Additionally, the greedy strategy involves only single-round vector additions that can be optimized via caching, making the practical complexity approximate to O(KZ). Compared with traditional client selection methods based on KL divergence minimization $O(2^{K}\cdot Z)$, this significantly reduces complexity and is more suitable for resource-constrained edge computing environments [28]. The lightweight heuristic in EDCS evaluates each candidate client’s contribution by estimating the change in information entropy on its local data, which serves as a proxy for data representativeness. This procedure involves only a small local forward pass and simple entropy computation, thus introducing negligible additional cost. The detailed process of EDCS is outlined in Algorithm 2.
**Algorithm 2.** EDCS**Input:** edge cluster n Candidate set $C_{n}$, label distribution $\left\{n_{c}\right\}$, participation rate ρ,random ratio λ
**Output:** Selected client set $S_{t}^{\left(n\right)}$ for edge cluster $E_{n}$ in round t
1: Set $K_{n}^{t}=\left\lceil \rho \cdot |C_{n}|\right\rceil$, $K_{n}^{t,\mathrm{rand}}=\left\lceil \lambda \cdot K_{n}^{t}\right\rceil$
2: $S_{t}^{(n),\mathrm{rand}}=RandomSample(C_{n},K_{n}^{t,\mathrm{rand}})$, $S_{t}^{(n),\mathrm{rem}}=C_{n}\backslash S_{t}^{(n),\mathrm{rand}}$
3: $n_{\mathrm{sum}}=[0,0,\ldots ,0]$    //Initialize accumulated label count vector4: for each client $k\in S_{t}^{(n),\mathrm{rand}}$ do5:     for z=1 to Z do6:         $n_{\mathrm{sum}}[z]+=n_{c}[z]$     // Accumulate label counts7:     end for8: end for9: Initialize $S_{t}^{(n),\mathrm{heur}}=\emptyset$
10: while $|S_{t}^{(n),\mathrm{heur}}|<K_{n}^{t}-K_{n}^{t,\mathrm{rand}}$ do11:     for each $k^{\prime }\in S_{t}^{(n),\mathrm{rem}}$ do12:         calculate merged entropy $H^{\mathrm{new}}$ using Equation (19)13:         Select client $k^{*}=\arg\;\max_{k^{\prime }\in S_{t}^{(n),\mathrm{rem}}}\left(H^{\mathrm{new}\;}-H(k^{\prime })\right)$
14:         Update: $S_{t}^{(n),\mathrm{heur}}=S_{t}^{(n),\mathrm{heur}}\cup \{k^{*}\}$, $n_{\mathrm{new}}=n_{\mathrm{sum}}+n_{k^{*}}$, $S_{t}^{(n),\mathrm{rem}}=S_{t}^{(n),\mathrm{rem}}\backslash \{c^{*}\}$
15:     end for16: end while17: return $S_{t}^{\left(n\right)}=S_{t}^{(n),\mathrm{rand}}\cup S_{t}^{(n),\mathrm{heur}}$


### 3.3. Hierarchical Asynchronous Dual-End Aggregation Mechanism

To enhance the adaptability and efficiency of federated training in edge heterogeneous environments, a hierarchical asynchronous collaborative training scheme is further proposed, which enables a flexible and efficient training process within the three-tier architecture of client-edge server-central server. The core of this mechanism lies in the following three aspects: First, the aggregation processes on the client side and server side are asynchronously decoupled, allowing the system to proceed in parallel and reduce waiting latency. Second, the introduction of a local aggregation mechanism within edge clusters helps improve training concurrency and alleviate centralization bottlenecks. Third, by setting the aggregation cycles for clients and servers, the global synchronization rhythm is uniformly controlled, ensuring the consistency of the global model and its convergence path.

At the beginning of each training round, taking the edge cluster $E_{n}$ as an example, clients determine partition points through the DAS module, each holding a front-end submodel $w_{c}^{n}$, while the edge server is responsible for the back-end submodel $w_{e}^{n}$.

In the t-th round, client $k\in S_{t}^{\left(n\right)}$ performs the following steps:

(1) Local mini-batch data $B_{k}$ is used to perform forward propagation through $w_{c}^{n}$ to generate activation values $h_{c}$;

(2) $h_{c}$ is sent to the edge server n, which completes the forward and backward propagation of the back-end model $w_{e}^{n}$

(3) To reduce communication frequency, the server caches $a_{c}$ and repeats training $\tau_{r}$ times;

(4) Backpropagation gradients are sent to the client to update its submodel $w_{c}^{n}$.

#### 3.3.1. Local Aggregation of Client Submodels

After every $\tau_{c}$ rounds of training, the edge server performs local aggregation on the client submodels within the cluster. Let client $C_{k}$ have a local sample size $D_{k}$, then its aggregation weight is defined as

(22) $$\alpha_{k}=\frac{D_{k}}{\sum_{C_{i}\in C_{n}}D_{i}}$$

The aggregation result of client submodels in the n-th edge cluster is

(23) $${\hat{w}}_{c}^{n}=\sum_{k\in C_{n}}\alpha_{k}\cdot w_{c}^{k}$$

The aggregated model ${\hat{w}}_{c}^{n}$ is broadcast to all $C_{k}\in C_{n}$ to synchronously update their local copies.

#### 3.3.2. Global Aggregation of Edge Server Submodels

After every $\tau_{e}$ rounds of training, the system performs global aggregation of edge server models. Let the total data volume of edge cluster $E_{n}$ be

(24) $$D_{n}=\sum_{C_{k}\in C_{n}}D_{k}$$

Its aggregation weight is

(25) $$\beta_{n}=\frac{D_{n}}{\sum_{i=1}^{N}D_{i}}$$

The corresponding global server model is

(26) $${\hat{w}}_{e}=\sum_{n=1}^{N}\beta_{n}\cdot w_{e}^{n}$$

This model is then delivered to all edge servers for the next training round.

Notably, the aggregation of client submodels and server submodels are independent and asynchronous in time, avoiding communication blockages caused by full synchronization. Define

(27) $$\tau_{G}=\mathrm{lcm}(\tau_{c},\tau_{e})$$

as the global synchronization period. When the number of training rounds reaches $t\mathrm{mod}\tau_{G}=0$, the system integrates the latest client submodels $\left\{{\hat{w}}_{c}^{n}\right\}$ and the global server model ${\hat{w}}_{e}$ to construct a complete global model by concatenation:

(28) $$W_{t}^{\mathrm{global}}=\mathrm{Combine}({\left\{{\hat{w}}_{c}^{n}\right\}}_{n=1}^{N},{\hat{w}}_{e})$$

This model is used for evaluation and inference in the current phase. Combining the design of the HiLo-Agg mechanism with the strategies in Algorithms 1 and 2, the overall training process of FLEX-SFL is outlined in Algorithm 3.

Although asynchronous aggregation can potentially introduce stale updates and increase gradient variance, HiLo-Agg mitigates these effects through its hierarchical design. Intra-cluster local aggregation at the edge servers reduces client-side noise before updates reach the cloud, the configurable client/edge/server aggregation cycles limit excessive staleness by controlling synchronization rhythm, and server-side caching with repeated local training further stabilizes updates by reducing dependence on single-round activations.
**Algorithm 3.** FLEX-SFL **Input:** Client set C, number of edge servers N, aggregation periods $\tau_{c}$, $\tau_{e}$, total global training rounds T, repeats training times $\tau_{r}$
**Output:** the global model $W_{t}^{\mathrm{global}}$ at global round t
1: $\tau_{G}=lcm(\tau_{c},\tau_{g})$, t=0    // Define global training period2: Initialize all model parameters $\left\{w_{c}^{k},w_{s}^{n}\right\}$
3: for τ = 1 to $\tau_{G}$ do4:     Invoke Algorithm 1(DAS) to determine partition points $P^{\mathrm{final}}$ and generate  model structures5:         Assign clients with identical structures to edge clusters $E=\{E_{1},E_{2},\ldots ,E_{N}\}$
6:         for each edge cluster $E_{n}\in E$ do7:             Invoke Algorithm 2 (EDCS) to select participant set $S_{t}^{\left(n\right)}$
8:             for each client $k\in S_{t}^{\left(n\right)}$ do9:                 Generate activation values $h_{c}=f(w_{c}^{k},B_{c})$
10:                 Upload $h_{c}$ to edge server $E_{n}$
11:                 edge Server-side $w_{e}^{n}$ repeats training $\tau_{r}$
12:                 Return gradients to update client model $w_{c}^{k}$
13:             end for14:         end for15:         if τ mod $\tau_{c}$ == 0 then16:             Aggregate all $w_{c}^{k}$ using Equation (23) to obtain ${\hat{w}}_{c}^{n}$, and broadcast to clients in the                  cluster17:         end if18:         if τ mod $\tau_{e}$ == 0 then19:             Aggregate all $w_{e}^{n}$ using Equation (26) to obtain ${\hat{w}}_{e}$, broadcast to all edge servers20:         end if21:         if τ mod $\tau_{G}$ == 0 then22:             Concatenate ${\hat{w}}_{e}$ and $\left\{{\hat{w}}_{c}^{n}\right\}$ using Equation (28) to generate $W_{t}^{\mathrm{global}}$
23:         end if24: end for25: t=t+1
26: return $W_{t}^{\mathrm{global}}$


## 4. Convergence Analysis

In this section, convergence analysis of the proposed FLEX-SFL framework is conducted.

**Assumption 1.** 
*(L-Smoothness):* *Each client’s local loss function* $F_{k}(w)$ *is *L*-smooth, i.e., there exists a constant, *L>0 *such that for all* x,y*,*

(29) $$F_{k}(y)\leq F_{k}(x)+\langle \nabla F_{k}(x),y-x\rangle +\frac{L}{2}\|y-x{\|}^{2}$$

**Assumption 2.** 
*(Unbiasedness and Bounded Variance of Stochastic Gradients): For the stochastic gradients *$g_{k}(w)$ *(of client *k*) and *$g_{n}(w)$* (of edge server *n*), the following conditions are satisfied: *

(30) $$\left\{\begin{array}{c} E[g_{k}(w)]=\nabla F_{k}(w)\\ \begin{array}{l} E[g_{n}(w)]=\nabla F_{n}(w)\\ E[\|g_{k}(w)-\nabla F_{k}(w){\|}^{2}]\leq \sigma_{k}^{2}\\ E[\|g_{n}(w)-\nabla F_{n}(w){\|}^{2}]\leq \sigma_{n}^{2} \end{array} \end{array}\right.$$

**Assumption 3.** 
*(Boundedness of Stochastic Gradients):*

(31) $$\left\{\begin{array}{l} E[\|g_{k}(w){\|}^{2}]\leq G^{2}\\ E[\|g_{n}(w){\|}^{2}]\leq G^{2} \end{array}\right.$$

**Lemma 1.** 
*(Local Training Error Squared Deviation Bound): Under Assumptions 1–3, if the number of local training steps is* $\tau_{1}$* and the learning rate satisfies *$\eta_{t}\leq \frac{1}{\sqrt{6L\tau_{1}}}$*, then for any communication round*t*, after continuously training*$\tau_{1}$*steps, the local model of client*k*(Lemma C.5 in Reference* [21]*):*

(32) $$\sum_{i=0}^{\tau -1}E\left[\|w_{k}^{t,i}-w_{k}^{t}{\|}^{2}\right]\leq 12\tau_{1}^{3}\eta_{t}^{2}\left(2\sigma_{k}^{2}+G^{2}\right)$$

**Theorem 1.** 
*Under the conditions of Assumptions 1–3 and Lemma 1, consider the FLEX-SFL framework with a general non-convex objective function. Let the number of update steps per round for clients and edge servers be* $\tau_{c}$* and *$\tau_{e}$*, respectively, the client participation rate be *ρ*, and a fixed learning rate *$\eta_{t}=\eta$* be adopted. After *T* rounds of iterations, the following convergence result holds:*

(33) $$\frac{1}{T}\sum_{t=0}^{T-1}E[\|\nabla f(w^{t}){\|}^{2}]\leq \frac{f(w^{0})-f^{*}}{\eta T(\tau_{c}+\tau_{e})}+\frac{C\eta }{\left(\tau_{c}+\tau_{e}\right)}$$

where $f^{*}=\underset{t}{\min}E[f(w^{t})]$, $w^{0}$ denotes the initial point, $\bar{\tau }=\max\{\tau_{c},\tau_{e}\}$, and C= $\sqrt{96}SG\sqrt{\sum_{k=1}^{K}a_{k}(2\sigma_{k}^{2}+G^{2})}{\bar{\tau }}^{2}+\frac{S}{2}\left(\sum_{k=1}^{K}\frac{a_{k}^{2}}{\rho }{\tau_{c}}^{2}(2\sigma_{k}^{2}+G^{2})+\sum_{n=1}^{N}a_{n}^{2}{\tau_{e}}^{2}(2\sigma_{n}^{2}+G^{2})\right)$.

**Proof of Theorem 1.** 
Take the full expectation of the smoothness inequality in Assumption 1:

(34) $$E[f(w^{t+1})]\leq E[f(w^{t})]+\underset{A}{\underset{︸}{E[\langle \nabla f(w^{t}),\Delta^{t}\rangle ]}}+\underset{B}{\underset{︸}{\frac{S}{2}E[\|\Delta^{t}{\|}^{2}]}}$$

where $\Delta^{t}=w^{t+1}-w^{t}$.

(1) For the second term A, by leveraging unbiasedness and converting it into the cumulative sum over all clients, we have:

(35) $$A=-\eta_{t}\cdot E\left[\langle \nabla f(w^{t}),\sum_{k\in S_{t}^{\left(n\right)}}\frac{a_{k}}{\rho }\sum_{i=0}^{\tau_{c}-1}g_{k}^{t,i}+\sum_{n=1}^{N}a_{n}\sum_{i=0}^{\tau_{e}-1}g_{n}^{t,i}\rangle \right]\;\;\;\;\;\;=-\eta_{t}\cdot E\left[\langle \nabla f(w^{t}),\sum_{k=1}^{K}a_{k}\left(\sum_{i=0}^{\tau_{c}-1}\nabla f_{k}(w_{k}^{t,i})+\sum_{i=0}^{\tau_{e}-1}\nabla f_{k}(w_{e}^{t,i})\right)\rangle \right]$$

For terms $\nabla f_{k}(w_{k}^{t,i})$ and $\nabla f_{k}(w_{k}^{t,i})$, the following holds:

(36) $$\left\{\begin{array}{l} \nabla f_{k}(w_{k}^{t,i})=\nabla f_{k}(w^{t})+\underset{\delta_{k}^{t,i}}{\underset{︸}{\left[\nabla f_{k}(w_{k}^{t,i})-\nabla f_{k}(w^{t})\right]}}\\ \nabla f_{k}(w_{e}^{t,i})=\nabla f_{k}(w^{t})+\underset{\delta_{e}^{t,i}}{\underset{︸}{\left[\nabla f_{k}(w_{e}^{t,i})-\nabla f_{k}(w^{t})\right]}} \end{array}\right.$$

After iterating over the above equation and performing decomposition on it, we have

(37) $$\left\{\begin{array}{l} \sum_{i=0}^{\tau_{c}-1}\nabla f_{k}(w_{k}^{t,i})=\tau_{c}\nabla f_{k}(w^{t})+\sum_{i=0}^{\tau_{c}-1}\delta_{k}^{t,i}\\ \sum_{i=0}^{\tau_{e}-1}\nabla f_{n}(w_{e}^{t,i})=\tau_{e}\nabla f_{k}(w^{t})+\sum_{i=0}^{\tau_{e}-1}\delta_{e}^{t,i} \end{array}\right.$$

Substitute Equation (39) into A; thus, A can be further expressed as

(38) $$A=-\eta_{t}\cdot E\left[\langle \nabla f(w^{t}),\underset{A_{1}}{\underset{︸}{\sum_{k=1}^{K}a_{k}(\tau_{c}+\tau_{e})\nabla f_{k}(w^{t})}}+\underset{A_{2}}{\underset{︸}{\sum_{k=1}^{K}a_{k}\left(\sum_{i=0}^{\tau_{c}-1}\delta_{k}^{t,i}+\sum_{i=0}^{\tau_{e}-1}\delta_{e}^{t,i}\right)}}\rangle \right]$$

(39) $$A_{1}=(\tau_{c}+\tau_{e})\nabla f_{k}(w^{t})$$

(40) $$\begin{array}{c} E\left[\|\nabla f(w^{t})\|\cdot \left\|A_{2}\right\|\right]=E\left[\|\nabla f(w^{t})\|\cdot \left\|\sum_{k=1}^{K}a_{k}\delta_{k}^{t}\right\|\right]\leq \sqrt{E\|\nabla f(w^{t}){\|}^{2}}\cdot \sqrt{E{\left\|\sum_{k=1}^{K}a_{k}\delta_{k}^{t}\right\|}^{2}}\\ \leq \\ G\cdot \sqrt{\sum_{k=1}^{K}a_{k}E\|\delta_{k}^{t}{\|}^{2}} \end{array}$$

where $\|\delta_{k}^{t}{\|}^{2}\leq 2\|\delta_{k,c}^{t}{\|}^{2}+2\|\delta_{k,e}^{t}{\|}^{2}$. Let $\bar{\tau }=\max\{\tau_{c},\tau_{e}\}$; meanwhile, by substituting the conclusion of Lemma 1, we further have

(41) $$\sum_{k=1}^{K}a_{k}E{\left\|\delta_{k}^{t}\right\|}^{2}\leq \sum_{k=1}^{K}4a_{k}E{\left\|\sum_{i=0}^{\bar{\tau }-1}\delta_{k}^{t,i}\right\|}^{2}\leq \sum_{k=1}^{K}4a_{k}\bar{\tau }\sum_{i=0}^{\bar{\tau }-1}\|\delta_{k}^{t,i}{\|}^{2}\leq \;\sum_{k=1}^{K}4a_{k}S^{2}\bar{\tau }\sum_{i=0}^{\bar{\tau }-1}\|(w_{k}^{t,i},w_{e}^{t,i})-w^{t}{\|}^{2}=\sum_{k=1}^{K}96a_{k}S^{2}{\bar{\tau }}^{4}\eta_{t}^{2}$$

Substitute the result of Equation (43) into Equation (42), and combine with the result of $A_{1}$; thus, we finally have

(42) $$A\leq -\eta_{t}(\tau_{1}+\tau_{2})E[\|\nabla f(w^{t}){\|}^{2}]+\sqrt{96}SG\sqrt{\sum_{k=1}^{K}a_{k}(2\sigma_{k}^{2}+G^{2})}\eta_{t}^{2}{\bar{\tau }}^{2}$$

(2) For the third term B, its expansion is given by

(43) $$B=\frac{S}{2}\eta_{t}^{2}E\left[{\left\|\underset{B_{1}}{\underset{︸}{\sum_{k\in S_{t}^{\left(n\right)}}\frac{a_{k}}{\rho }\sum_{i=0}^{\tau_{c}-1}g_{k}^{t,i}}}+\underset{B_{2}}{\underset{︸}{\sum_{n=1}^{N}a_{n}\sum_{i=0}^{\tau_{e}-1}g_{n}^{t,i}}}\right\|}^{2}\right]$$

After performing processing on $B_{1}$ and $B_{2}$, we have

(44) $$E[\|B_{1}{\|}^{2}]\leq \sum_{k=1}^{K}\frac{a_{k}^{2}}{\rho }{\tau_{c}}^{2}(2\sigma_{k}^{2}+G^{2})$$

(45) $$E[\|B_{2}{\|}^{2}]\leq \sum_{n=1}^{N}a_{n}^{2}{\tau_{e}}^{2}(2\sigma_{e}^{2}+G^{2})$$

Considering independence, Term $E[\langle \sum_{k\in S_{t}^{\left(n\right)}}\frac{a_{k}}{\rho }\sum_{i=0}^{\tau_{c}-1}g_{k}^{t,i},\sum_{n=1}^{N}a_{n}\sum_{i=0}^{\tau_{e}-1}g_{n}^{t,i}\rangle ]$ is negligible. By substituting the results of Equations (44) and (45) into Equation (43), we thus obtain the expression for Term B as follows:

(46) $$B\leq \frac{S}{2}\eta_{t}^{2}\left(\sum_{k=1}^{K}\frac{a_{k}^{2}}{\rho }{\tau_{c}}^{2}(2\sigma_{k}^{2}+G^{2})+\sum_{n=1}^{N}a_{n}^{2}{\tau_{e}}^{2}\left(2\sigma_{n}^{2}+G^{2}\right)\right)$$

(3) Substitute the relational inequalities satisfied by Term A and Term B into the initial expression (34); thus, we have

(47) $$E[f(w^{t+1})]\;\leq E[f(w^{t})]-\eta_{t}(\tau_{1}+\tau_{2})E[\|\nabla f(w^{t}){\|}^{2}]+C\eta_{t}^{2}$$

where $C=\sqrt{96}SG\sqrt{\sum_{k=1}^{K}a_{k}(2\sigma_{k}^{2}+G^{2})}{\bar{\tau }}^{2}+\frac{S}{2}\left(\sum_{k=1}^{K}\frac{a_{k}^{2}}{\rho }{\tau_{c}}^{2}(2\sigma_{k}^{2}+G^{2})+\sum_{n=1}^{N}a_{n}^{2}{\tau_{e}}^{2}\left(2\sigma_{n}^{2}+G^{2}\right)\right)$.

Meanwhile, by summing the above equation on both sides for t=0,…,T−1 respectively, we have

(48) $$\sum_{t=0}^{T-1}E[f(w^{t+1})]\leq \sum_{t=0}^{T-1}E[f(w^{t})]-(\tau_{c}+\tau_{e})\sum_{t=0}^{T-1}\eta_{t}E[\|\nabla f(w^{t}){\|}^{2}]+C\sum_{t=0}^{T-1}\eta_{t}^{2}$$

Further perform the method of splitting terms and canceling out

(49) $$E[f(w^{T})]-E[f(w^{0})]\leq -(\tau_{c}+\tau_{e})\sum_{t=0}^{T-1}\eta_{t}E[\|\nabla f(w^{t}){\|}^{2}]+C\sum_{t=0}^{T-1}\eta_{t}^{2}$$

Let $f^{*}=\underset{t}{\min}E[f(w^{t})]$ and $f^{0}=E[f(w^{0})]$. Since $f(w^{T})\geq f^{*}$, by transforming the above equation, we have

(50) $$\frac{1}{\sum_{t=0}^{T-1}\eta_{t}}\sum_{t=0}^{T-1}\eta_{t}E[\|\nabla f(w^{t}){\|}^{2}]\leq \frac{f^{0}-f^{*}}{(\tau_{c}+\tau_{e})\sum_{t=0}^{T-1}\eta_{t}}+\frac{C\sum_{t=0}^{T-1}\eta_{t}^{2}}{(\tau_{c}+\tau_{e})\sum_{t=0}^{T-1}\eta_{t}}$$

When the learning rate is set to $\eta_{t}=\eta$, we finally obtain

(51) $$\frac{1}{T}\sum_{t=0}^{T-1}E[\|\nabla f(w^{t}){\|}^{2}]\leq \frac{f^{0}-f^{*}}{\eta T(\tau_{c}+\tau_{e})}+\frac{C\eta }{\left(\tau_{c}+\tau_{e}\right)}$$

Equation (51) has conformed to the expression form of Theorem 1, and the proof is completed. □

By analyzing the result of Equation (51), it can be concluded that the FLEX-SFL framework can ultimately achieve a function value convergence rate of the O(1/T) under non-convex conditions. Furthermore, compared with random client selection, the EDCS mechanism in FLEX-SFL achieves a higher score in terms of data distribution diversity. Under the same conditions, this mechanism leads to a higher participation rate ρ, a smaller stochastic gradient bias $\sigma_{k}$, and a smaller upper bound G; consequently, the upper bound of the actual final convergence error will be smaller.

## 5. Experiments

This section aims to evaluate the performance of the proposed FLEX-SFL framework in typical edge heterogeneous environments and validate its adaptability and efficiency advantages under the dual challenges of statistical heterogeneity and system heterogeneity. All experiments were conducted on a local computing platform equipped with an AMD Ryzen 9 7940HX CPU and an NVIDIA RTX 4070 GPU, running the Windows 11 operating system, within the programming environment of Python 3.9 and the deep learning framework of PyTorch 1.13.

### 5.1. Datasets and Configurations

To systematically evaluate the adaptability of FLEX-SFL under different task complexities and heterogeneous environments, three widely used image classification datasets were selected: FMNIST, CIFAR-10, and CIFAR-100. These datasets cover grayscale and color images, low-dimensional and high-dimensional features, and multi-class and fine-grained tasks, making them representative and challenging. The details are as follows:

1. FMNIST: A grayscale image dataset consisting of 10 classes of fashion items with 28 × 28 pixel size, used for medium- to low-complexity image recognition tasks.
2. CIFAR-10: Contains 10 classes of 32 × 32 color images (e.g., airplanes, cars, cats), used for general object classification.
3. CIFAR-100: Structurally similar to CIFAR-10 but with 100 finer classes, suitable for evaluating modeling capabilities in high-dimensional multi-class scenarios.

The model structures and training parameters configured for different datasets are shown in Table 2. FMNIST uses a lightweight convolutional neural network (2 convolutional layers + 2 fully connected layers), while CIFAR-10 and CIFAR-100 adopt VGG-16 and VGG-19, respectively, to adapt to their complexity differences. The datasets and configurations used in the experiment are shown in Table 2.

A total of 100 clients were simulated, with the client participation rate set to ρ=0.2 (i.e., 20 clients were randomly selected for training in each round), and the number of edge servers was 3 to support client cluster aggregation under heterogeneous partitioning. The following are the corresponding hyperparameter settings in the HiLo-Agg mechanism:

- Local training and aggregation: Clients perform local aggregation within edge clusters after every $\tau_{c}=4$ rounds of local training.
- Global aggregation of edge servers: Global aggregation of edge server submodels is performed every $\tau_{e}=2$ rounds.
- Server-side caching mechanism: After receiving client activation values, edge servers repeatedly train $\tau_{r}=10$ times to reduce communication frequency (i.e., the server updates the model 10 times using the same activation values in each communication round).

Additionally, to enhance heterogeneity, two types of strategies were designed for the experiments:

(a) Label distribution heterogeneity: Each client contained only 2 classes (FMNIST/CIFAR-10) or 20 classes (CIFAR-100) to simulate the locality bias in data collection.

(b) Data quantity imbalance: Client sample sizes followed a power-law distribution, reflecting the typical phenomenon of uneven data distribution in real-world terminals.

### 5.2. Comparative Methods 

To comprehensively evaluate the effectiveness of FLEX-SFL in heterogeneous edge scenarios, six representative federated optimization methods were selected as comparison baselines, covering classic FL and SFL paradigms. The performance of each method on different datasets is shown in the table below:

(1) FedAvg: As a standard Federated Learning (FL) method, this method completes collaborative modeling through local training and parameter averaging, but is vulnerable to client drift in heterogeneous scenarios.

(2) FedProx: On the basis of FedAvg, this method introduces a regularized constraint term to mitigate model deviation caused by non-IID (non-independently and identically distributed) data and improves convergence stability under statistical heterogeneity. In this paper, the coefficient of the proximal term is set to 0.3.

(3) MOON (Model-Contrastive Federated Learning): Integrating the idea of contrastive learning, this method alleviates model bias under heterogeneous data distribution by enhancing the consistency of representation between local and global models.

(4) SplitFed: A typical synchronous communication Split Learning framework, this method splits the model into two parts, which possesses both the resource adaptability of SL and the distributed advantages of FL, but lacks a client selection mechanism.

(5) SplitMix (Split Mixing) [22]: This method supports customized model splitting according to requirements, allowing clients to flexibly splice local submodels from multiple modules and enabling per-round reconstruction. However, it lacks an asynchronous communication mode.

(6) FedRich [29]: This is a state-of-the-art SFL framework that clusters clients based on capability metrics (e.g., computing power, bandwidth) and selects clients by calculating the loss between the selected data distribution and the edge server’s data distribution.

To ensure fair comparison, all methods involving model partitioning (SplitFed, SplitMix, FedRich, and FLEX-SFL) adopt a unified partitioning strategy. The first 1–3 convolutional modules are designated as client-side submodels, with remaining layers as edge server submodels. This configuration standardizes model partitioning positions, eliminating interference from partitioning strategy differences and focusing on core distinctions in aggregation mechanisms, client selection, and heterogeneous adaptability.

### 5.3. Experimental Results

#### 5.3.1. Performance Across Different Datasets

To comprehensively evaluate the applicability and advantages of the proposed FLEX-SFL framework in various heterogeneous task scenarios, systematic tests were conducted on three datasets—FMNIST, CIFAR-10, and CIFAR-100—comparing FLEX-SFL with six existing baseline methods. In FLEX-SFL’s heuristic selection mechanism, the random selection ratio was set to λ=0.4 to balance diversity and representativeness, with all methods trained under consistent configurations to ensure fair comparison.

**Figure 4:**
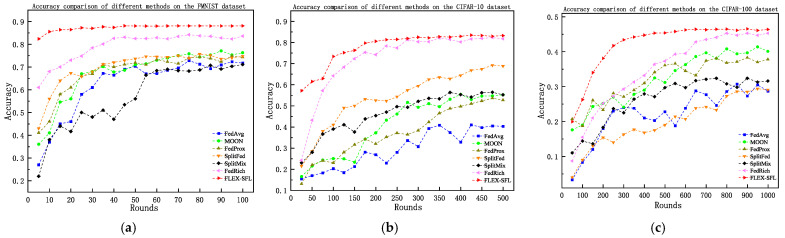
The accuracy variation of different methods across different datasets. (**a**) FMNIST; (**b**) CIFAR-10; (**c**) CIFAR-100.

Table 3 lists the test accuracies of each method after 100, 500, and 1000 training rounds, while Figure 4 illustrates the accuracy trends with training rounds. The results show that FLEX-SFL achieved optimal performance on all three datasets, validating its generalization capability in both system and statistical heterogeneous environments.

On the FMNIST dataset, FLEX-SFL achieved a final accuracy of 88.1%, outperforming traditional federated learning methods—FedAvg (71.4%), FedProx (73.6%), and MOON (76.3%)—by 16.7, 14.5, and 11.8 percentage points (pp), respectively, and surpassing Split Learning (SL)-based methods SplitFed (74.7%) and SplitMix (71.1%) (which suffer from static model partitioning and a synchronous communication-induced inability to handle device computing power disparities) by 13.4 and 17 pp. Even compared with the state-of-the-art (SOTA) method FedRich (83.6%), FLEX-SFL still maintained a 4.5 percentage point (pp) advantage in final accuracy.. On the CIFAR-10 dataset, FLEX-SFL led all methods, with 83.8% accuracy (a 2.1 pp improvement over FedRich (81.7%)). Traditional FL methods (FedAvg, FedProx) yield accuracies below 53% due to their incompetence in processing highly heterogeneous data, while SplitFed (68.8%), though validating model partitioning effectiveness, faces communication bottlenecks from synchronous aggregation. By contrast, FLEX-SFL enhances training stability via entropy-driven client selection (to expand sample diversity coverage) and hierarchical asynchronous aggregation (to reduce latency). In the high-complexity CIFAR-100 scenario (characterized by fine-grained categories and extremely unbalanced data distribution), FLEX-SFL still outperformed all methods, with 46.4% accuracy (1.5 pp higher than FedRich (44.9%)), whereas traditional methods exhibited severe performance degradation (e.g., SplitFed only reached 29.1%). FLEX-SFL addresses high-dimensional heterogeneity challenges through device-aware adaptive segmentation (for balancing computing loads) and edge cluster-based local aggregation (for enhancing model consistency).

#### 5.3.2. Comparison of Convergence Rates

To further verify the engineering feasibility and execution efficiency of FLEX-SFL in practical deployment scenarios, this section takes the target accuracy from Section 5.3.1 as the benchmark (setting thresholds of 70% for FMNIST, 50% for CIFAR-10, and 30% for CIFAR-100) and compares FLEX-SFL with representative split federated learning methods (SplitFed, SplitMix, FedRich, etc.) in terms of training rounds and cumulative running time, with results shown in Table 4.

On FMNIST, FLEX-SFL reached 70% accuracy in only 3 communication rounds (90.9% fewer than SplitFed (33 rounds), 76.9% fewer than FedRich (13 rounds)) and took 10.27 s (86.4% shorter than SplitFed (75.57 s), 92.8% shorter than SplitMix (143.39 s), 56.1% shorter than FedRich (23.36 s)), with this advantage stemming from device-aware adaptive segmentation, which assigns lightweight submodels (e.g., the first two convolutional layers) to low-computing-power devices and complex layers to high-computing-power ones. In the CIFAR-10 task, it achieved 50% accuracy in 17 rounds (85.0% fewer than SplitFed (113 rounds), 74.2% fewer than FedRich (66 rounds)) and ran for 22.16 s (88.7% shorter than SplitFed (195.32 s), 96.4% shorter than SplitMix (623.43 s), 91.5% shorter than FedRich (262.13 s)), driven by entropy-driven client selection (screening representative clients via label entropy to reduce local deviation) and hierarchical asynchronous aggregation (avoiding full-synchronous communication blockages). On the high-complexity CIFAR-100, FLEX-SFL hit 30% accuracy in 118 rounds (87.4% fewer than SplitFed (938 rounds), 62.8% fewer than FedRich (317 rounds)) and took 243.33 s (86.3% shorter than SplitFed (1781.52 s), 71.0% shorter than SplitMix (836.51 s), 73.4% shorter than FedRich (913.26 s)), benefiting from edge cluster partitioning (reducing cross-cluster communication via intra-cluster aggregation) and server-side caching (reusing activation values to boost single-round effective computation by 10×).

#### 5.3.3. Resource Consumption

(1) Theoretical Analysis

The FLEX-SFL framework is based on the split federated learning paradigm, offloading the computational and storage burdens of deep models to edge servers. Therefore, resource consumption only needs to consider the communication and computational overhead on the client side.

- Communication Overhead Analysis:

Communication overhead consists of two components: submodel parameter transmission and feature activation value transmission. Submodel Parameter Transmission Overhead: Clients upload gradients and download aggregated models every $\tau_{c}$ rounds (intra-cluster aggregation period). The single-client overhead per transmission is $\tau_{G}/\tau_{c}\times 2\beta_{k}|W|$ (where $\beta_{k}$ is the submodel proportion and |W| is the size of the full model parameters). Thus, the total overhead for all clients in the system is $2\rho \cdot \tau_{G}/\tau_{c}\sum_{n=1}^{N}\beta_{n}|W||U_{n}|$, where $\beta_{n}$ is the average submodel proportion of the n-th cluster, and $\left|U_{n}\right|$ is the number of clients in the cluster.

Feature Activation Value Transmission Overhead: In each local round, clients need to upload activation values. The single-client overhead is $2PD_{k}Q_{k}$ (where P is the sampling rate, and $Q_{k}$ is the feature dimension). The total overhead for all clients in a global round is $2\rho \tau_{G}P\sum_{n=1}^{N}\sum_{i=1}^{\left|U_{n}\right|}D_{u_{i}}Q_{n}$.

Summing the two components, the total communication resource consumption in one global round is $2\rho \sum \left(\tau_{G}/\tau_{c}\cdot \beta_{n}|W||U_{n}|+P\tau_{G}\sum D_{u_{i}}Q_{n}\right)$.

- Computational Overhead Analysis:

Computational overhead only considers the local training load on clients. Assuming the computation consumption for a client to train a full model is ν, and the computation amount for each local round is $\beta_{k}\tau_{G}\nu$, the total computational overhead for all clients in one round is $\rho \tau_{G}\nu \sum_{n=1}^{N}\beta_{n}\left|U_{n}\right|$.

(2) Experimental Analysis

To evaluate the efficiency of FLEX-SFL in terms of resource usage, we conducted a comprehensive comparison with three representative split learning-based frameworks: SplitFed, SplitMix, and FedRich. All methods share consistent experimental configurations, and for FLEX-SFL, the local aggregation interval $\tau_{c}=4$ and the global aggregation interval $\tau_{e}=2$ were used, following the default setup in FedRich [29], to ensure fairness.

- Per-Round Communication and Computation Costs

Table 5 (upper half) presents the average communication and computation overhead per global round across three datasets. For communication cost, FedRich achieved the lowest transmission overhead due to its lightweight client–server interaction design, consuming only 0.76 MB per round on FMNIST. In contrast, FLEX-SFL incurred higher communication costs (e.g., 2.96 MB on FMNIST), approximately 3.9× that of FedRich. This increase stems from the HiLo-Agg architecture in FLEX-SFL, which introduces additional intra-cluster transmissions and repeated server-side computations to enable asynchronous training and mitigate straggler effects.

However, FLEX-SFL significantly reduces the client-side computation burden due to its dynamic segmentation (DAS) and edge offloading mechanism. On all datasets, FLEX-SFL exhibited the lowest per-round computation cost, e.g., 2.43 MFLOPs on FMNIST, outperforming FedRich (2.7 MFLOPs) and SplitFed (3.81 MFLOPs). This efficiency gain is primarily attributed to the adaptive submodel allocation, which assigns lighter computational loads to resource-constrained clients while delegating heavier components to edge servers.

- Total Resource Consumption to Reach Target Accuracy

The lower part of Table 5 reports the total communication and computation cost required for each method to reach the predefined accuracy thresholds: 70% for FMNIST, 50% for CIFAR-10, and 30% for CIFAR-100.

Thanks to its faster convergence rate, FLEX-SFL achieved substantial savings in overall resource consumption. On FMNIST, it only required 8.81 MB in communication and 7.29 MFLOPs in computation to reach 70% accuracy, which are 10.1% and 79.2% lower than the requirements for FedRich, respectively. Similar trends were observed on CIFAR-10 and CIFAR-100. Although the per-round communication overhead of FLEX-SFL is higher, the reduced number of required training rounds significantly offsets this cost. For example, on CIFAR-100, FLEX-SFL completed the task in 118 rounds, whereas FedRich took 317 rounds, leading to a 48.5% reduction in total communication (3044.4 MB vs. 6041.7 MB estimated if scaled) and a 67.9% reduction in computation (3406.7 MFLOPs vs. 10616.3 MFLOPs).

These results demonstrate that FLEX-SFL, despite higher per-round transmission, achieved superior overall efficiency due to its enhanced convergence behavior and adaptive training mechanisms.

### 5.4. Hyperparameter Impact Exploration

This subsection investigates the impact of hyperparameters on the FLEX-SFL framework, focusing on the aggregation intervals $\left(\tau_{c},\tau_{e}\right)$ of the HiLo-Agg mechanism and the random selection ratio (λ) of the EDCS strategy. Experiments were conducted on the FMNIST dataset with 100 training rounds, adjusting one variable at a time to record test accuracy and quantify the influence of parameters on convergence efficiency and model performance.

#### 5.4.1. Aggregation Intervals $\tau_{c}$ and $\tau_{e}$

FLEX-SFL’s hierarchical asynchronous aggregation mechanism achieves a dynamic balance between communication efficiency and model consistency by adjusting the client aggregation period $\left(\tau_{c}\right)$ and edge server aggregation period $\left(\tau_{e}\right)$. Grid search was performed on the FMNIST dataset $\left(\tau_{c},\tau_{e}\in \{1,2,\ldots ,8\}\right)$, with the random selection ratio fixed at λ=0.4, and the average accuracy of the last 10 rounds recorded as the performance metric (Table 6).

Horizontal analysis: When $\tau_{c}=4$, as $\tau_{e}$ increased from 1 to 8, accuracy decreased from 0.8941 to 0.8344, a drop of 5.97%. This indicates that reducing edge server aggregation frequency leads to delayed global model updates and insufficient information fusion, consistent with the theoretical conclusion in the convergence analysis that “aggregation delay terms dominate the error upper bound” (Theorem 1). Therefore, shortening $\tau_{e}$ can effectively improve model synchronization efficiency and suppress error accumulation.

Vertical comparison: Under $\tau_{g}=1$, as $\tau_{c}$ increased from 1 to 8, accuracy first increased then decreased (0.8814→0.8941→0.8923). Moderate increases in $\tau_{c}$ enhance model expressiveness through local training, but excessive prolongation amplifies local bias and noise. Experiments showed that the optimal accuracy (0.8941) was achieved when $\tau_{c}=4$ and $\tau_{e}=1$. When $\tau_{c}=1$ and $\tau_{e}=1$, this parameter setting is equivalent to synchronous hierarchical aggregation—specifically, it corresponds to the ablation experiment where the HiLo-Agg mechanism was not used. The accuracy under this setting was 0.8814, which is 1.27 percentage points lower than that of the optimal combination. This verifies the effectiveness of the asynchronous decoupled aggregation mechanism.

Thus, optimizing aggregation intervals requires balancing local training depth and global information synchronization. Combinations of shorter $\tau_{e}$ (e.g., 1–2 rounds) and moderate $\tau_{c}$ (e.g., 4 rounds) significantly enhance FLEX-SFL’s performance.

#### 5.4.2. Random Participation Ratio λ

FLEX-SFL’s EDCS module employs a two-stage client selection mechanism of “random sampling + entropy-driven screening,” where the random participation ratio λ determines the balance between candidate set diversity and heuristic optimization space. A larger value of λ ensures the initial candidate set covers more types of devices, aiding in capturing global features of data distributions but compressing the optimization space for entropy screening in the second stage. A smaller λ focuses the candidate set on high-entropy samples, improving screening accuracy but potentially reducing sample diversity.

To quantify the impact of λ on model performance, experiments were conducted on the FMNIST dataset with λ∈{0.2,0.4,0.6,0.8,1.0}, fixing $\tau_{c}=4$, $\tau_{e}=2$, and selecting seven clients per edge cluster per round. When λ=1, the selection strategy degenerated to pure random sampling, equivalent to an ablation experiment disabling EDCS.

**Figure 5:**
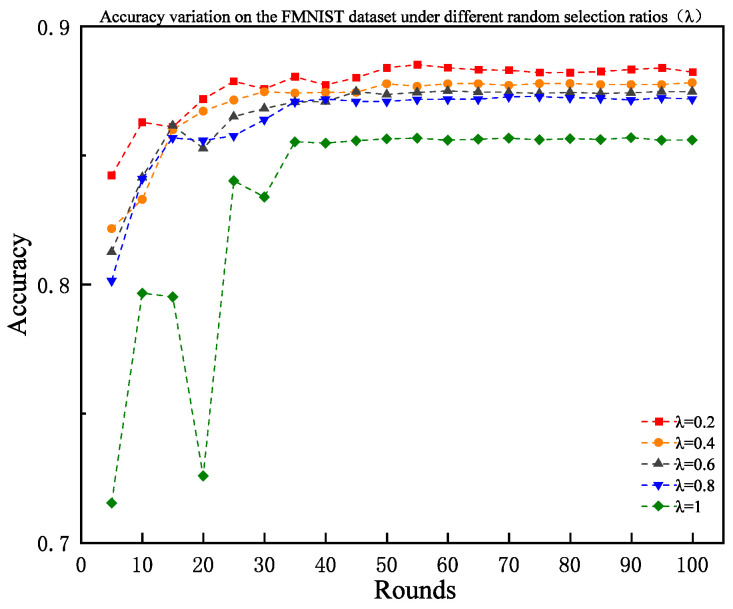
Accuracy variation under different random selection ratios λ on the FMNIST dataset.

Figure 5 illustrates the test accuracy trends under different λ, with final results of 0.8823, 0.8782, 0.8747, 0.8719, and 0.856, respectively. The results show that completely random selection (λ=1) yielded the worst performance, at 0.856, while combinations of lower randomness (λ=0.2) and high-proportion entropy-driven screening enhanced sample quality while maintaining data diversity, balancing client selection efficiency and accuracy. These findings provide a basis for parameter configuration in practical applications, advocating “priority for heuristic screening with moderate randomness retention.”

## 6. Conclusions

To address the critical challenges of device heterogeneity, statistical non-IID data, and communication inefficiency in edge intelligent systems, this paper proposes FLEX-SFL, a flexible and efficient optimization framework for split federated learning. Centered around three core aspects—adaptive model structuring, representative client selection, and hierarchical asynchronous aggregation—FLEX-SFL integrates a device-aware adaptive segmentation (DAS) strategy, an entropy-driven client selection (EDCS) mechanism, and a hierarchical local asynchronous aggregation (HiLo-Agg) scheme to enable collaborative optimization under multidimensional heterogeneity.

From a theoretical perspective, the convergence analysis results confirm that FLEX-SFL achieves favorable global convergence guarantees under non-convex objectives, and further reveals how parameters such as participation ratio and local training steps influence the convergence upper bound. Empirical results on diverse non-IID datasets, including FMNIST, CIFAR-10, and CIFAR-100, demonstrate that FLEX-SFL consistently outperforms existing state-of-the-art approaches in terms of model accuracy, convergence speed, and resource efficiency, showcasing strong adaptability and deployment viability in heterogeneous edge scenarios.

Future work will focus on extending FLEX-SFL to heterogeneous IoT edge platforms, such as Raspberry Pi and NVIDIA Jetson clusters, to experimentally validate its scalability and robustness under real-world resource-constrained conditions. Furthermore, we plan to investigate its integration with advanced paradigms, including federated distillation and self-supervised learning for broader applications in industrial IoT and intelligent healthcare.

## Figures and Tables

**Table 1:** Summary of notation.

Notation	Description	Notation	Description
K	the number of clients	$\pi_{k}$	the label distribution vector of client *k*
N	the number of edge servers	$K_{n}^{t}$	the number of selected clients in edge cluster *n* at round *t*
V	the number of neural network layers	$S_{t}^{\left(n\right)}$	the client set selected in edge cluster n at round *t*
C	the set of all clients	$S_{t}^{(n),\mathrm{rand}}$	the randomly selected client set in edge cluster *n* at round *t*
P	the set of cutting points for all clients	$S_{t}^{(n),\mathrm{rem}}$	the client pool for selection in edge cluster n at round t
λ	the client selection ratio in the stochastic phase	$w_{e}^{n}$	the submodel of the edge server in edge cluster *n*
$R_{k}$	the client *k*	${\hat{w}}_{c}^{n}$	the submodel after client aggregation in edge cluster *n*
$p_{k}$	the model cutting points of client *k*	${\hat{w}}_{s}$	the model after aggregating edge server submodels
$\delta_{k}$	the load factor of client *k*	$W_{T}^{\mathrm{global}}$	the global model at global round *T*
$w_{c}^{k}$	the submodel of client *k*	$\tau_{c}$	the client submodel aggregation period
$D_{k}$	the amount of data owned by client *k*	$\tau_{e}$	the redundant propagation times of edge server submodels
$E_{n}$	the *n*-th edge cluster	$\tau_{G}$	the local training period in a global round
$H_{k}$	the information entropy for client *k*	$\eta_{t}$	the learning rate at round *t*

**Table 2:** Datasets and configurations used in the experiment.

Dataset	Model	Learning Rate	Decay Rate	Classes per Client
FMNIST	2 conv +2 fc	0.1	0.98	2
CIFAR-10	VGG-16	0.03	0.997	2
CIFAR-100	VGG-19	0.03	0.998	20

**Table 3:** The final accuracy of different methods on the FMNIST, CIFAR-10, and CIFAR-100 datasets.

Method	Dataset		
	FMNIST	CIFAR-10	CIFAR-100
FedAvg	71.7%	40.3%	28.7%
FedProx	74.3%	52.7%	37.8%
MOON	76.3%	55.2%	40.1%
SplitFed	74.7%	68.8%	29.1%
SplitMix	71.1%	55.2%	31.6%
FedRich	83.6%	81.7%	45.3%
FLEX-SFL	88.1%	83.2%	46.4%

**Table 4:** Comparison of communication rounds (round) and actual running time (s) for different split federated methods to reach threshold accuracy on FMNIST, CIFAR-10, and CIFAR-100.

Method	FMNIST		CIFAR-10		CIFAR-100	
	Rounds	Times	Rounds	Times	Rounds	Times
SplitFed	33	75.57	113	195.32	938	1781.52
SplitMix	68	143.39	269	623.43	506	836.51
FedRich	13	23.36	66	262.13	317	913.26
FLEX-SFL	3	10.27	17	22.16	118	243.33

**Table 5:** Per-round and cumulative communication and computation costs to reach target accuracy on three datasets.

**Method**	**Comms. (MB)**			**Comps. (MFLOPs)**		
	**FMNIST**	**CIFAR-10**	**CIFAR-100**	**FMNIST**	**CIFAR-10**	**CIFAR-100**
SplitFed	1.21	7.65	7.65	3.81	39.78	39.78
SplitMix	0.98	7.32	7.32	3.32	36.72	36.72
FedRich	0.76	6.45	6.45	2.7	33.49	33.49
FLEX-SFL	2.96	25.8	25.8	2.43	28.87	28.87
**Method**	**Total Comms. (MB)**			**Total Comps. (MFLOPs)**		
	**FMNIST**	**CIFAR-10**	**CIFAR-100**	**FMNIST**	**CIFAR-10**	**CIFAR-100**
SplitFed	39.3	864.45	7175.7	125.73	4495.1	37,313.6
SplitMix	66.64	1969.1	3704.1	225.8	9877.7	18,580.3
FedRich	9.88	425.7	6041.7	35.1	2210.3	10,616.3
FLEX-SFL	8.81	438.6	3044.4	7.29	490.7	3406.7

**Table 6:** Accuracy under different aggregation frequencies $\tau_{c}$ and $\tau_{e}$ on the FMNIST dataset.

$\tau_{c}$	$\tau_{e}$							
	1	2	3	4	5	6	7	8
1	0.8814	0.8788	0.8655	0.8606	0.8572	0.8501	0.8461	0.8434
2	0.8864	0.8813	0.8686	0.8641	0.8612	0.8467	0.8449	0.8403
3	0.8927	0.8788	0.8714	0.8638	0.8494	0.8589	0.8495	0.8426
4	0.8941	0.8794	0.8732	0.8651	0.8500	0.8433	0.8432	0.8344
5	0.8897	0.8819	0.8687	0.8637	0.8613	0.8421	0.8435	0.8382
6	0.8902	0.8847	0.8726	0.8626	0.8618	0.8533	0.8484	0.8442
7	0.8897	0.8792	0.8666	0.8648	0.8484	0.8505	0.8446	0.8424
8	0.8923	0.8780	0.8731	0.8674	0.8580	0.8403	0.8520	0.8385

## Data Availability

Data are contained within the article.
